# Upfront Versus Deferred CDK4/6 Inhibitor Use in Hormone Receptor-Positive, HER2-Negative Metastatic Breast Cancer: A Real-World Study

**DOI:** 10.3390/biomedicines14091881

**Published:** 2026-08-23

**Authors:** Pınar Kubilay Tolunay, Bediz Kurt İnci, Berkan Karabuğa, Ali Topkaç, Şura Usta, Ergin Aydemir, Nejat Emre Öksüz, Enes Erul, İrem Küçükşahin, Hatime Arzu Yaşar, Hakan Akbulut, Ülkü Yalçıntaş Arslan, Öztürk Ateş

**Affiliations:** 1 Department of Medical Oncology, Ankara University School of Medicine, Ankara 06590, Turkey; nejatemreoksuz@gmail.com (N.E.Ö.); eneserul@hotmail.com (E.E.); arzuyasar@gmail.com (H.A.Y.); hakan_akbulut30@yahoo.com (H.A.); 2 Department of Medical Oncology, Dr. Abdurrahman Yurtaslan Oncology Training and Research Hospital, University of Health Sciences, Ankara 06200, Turkey; bedizkurt@gmail.com (B.K.İ.); drbkarabuga@gmail.com (B.K.); dralitopkac@gmail.com (A.T.); sura.oztekin@hotmail.com (Ş.U.); draydemirergin@gmail.com (E.A.); dr_iremoner@hotmail.com (İ.K.); ulkuarslan63@gmail.com (Ü.Y.A.); dr.ozturkates@gmail.com (Ö.A.)

**Keywords:** CDK4/6 inhibitors, metastatic breast cancer, hormone receptor-positive breast cancer, treatment sequencing, first-line treatment, second-line treatment, overall survival, real-world data

## Abstract

**Background/Objectives**: Cyclin-dependent kinase 4/6 inhibitors (CDK4/6i) are commonly used as first-line treatment for hormone receptor-positive, human epidermal growth factor receptor 2 (HER2)-negative metastatic breast cancer. However, the optimal timing of CDK4/6i initiation remains uncertain. This study compared overall survival (OS) between upfront first-line and deferred second-line CDK4/6i use in routine clinical practice. **Methods**: This retrospective, two-center cohort study included 403 patients with hormone receptor-positive, HER2-negative metastatic breast cancer who received a CDK4/6i between January 2019 and December 2024. Patients were categorized into upfront-use and deferred-use groups according to whether CDK4/6i treatment was initiated in the first or second metastatic treatment line. Overall survival was estimated using the Kaplan–Meier method. An exploratory analysis of progression-free survival (PFS) from CDK4/6i initiation was also performed. Cox proportional hazards regression, inverse probability of treatment weighting (IPTW), and a 12-month landmark analysis were performed. **Results**: Of 403 patients, 311 received upfront treatment and 92 received deferred treatment. At a median follow-up of 63.0 months, the deferred-use group had higher rates of visceral metastasis and endocrine-resistant disease. Median PFS from CDK4/6i initiation was longer in the upfront-use group than in the deferred-use group (27.0 vs. 14.0 months; log-rank *p* < 0.001). Median OS was 61.0 months in the upfront-use group and 76.0 months in the deferred-use group, without a statistically significant difference (log-rank *p* = 0.059). After adjustment for clinical factors and year of metastatic diagnosis, deferred use was not independently associated with OS (adjusted hazard ratio [HR] 1.167, 95% confidence interval [CI] 0.772–1.765, *p* = 0.463). Similar results were obtained in the IPTW analysis (HR 0.925, 95% CI 0.552–1.549; *p* = 0.766) and the 12-month landmark analysis. **Conclusions**: Deferred second-line CDK4/6i use was not independently associated with inferior overall survival compared with upfront use. These findings support an individualized, risk-adapted sequencing approach in carefully selected patients but do not establish equivalence or justify routine deferral.

## 1. Introduction

The management of hormone receptor-positive (HR-positive), human epidermal growth factor receptor 2-negative (HER2-negative) metastatic breast cancer has changed substantially with the integration of cyclin-dependent kinase 4 and 6 inhibitors (CDK4/6i) into endocrine-based treatment. In randomized phase III trials, these agents consistently prolonged progression-free survival (PFS) when combined with aromatase inhibitors in the first-line setting or with fulvestrant after progression on prior endocrine therapy [1,2,3,4,5,6,7]. Overall survival (OS) benefit has also been reported in several studies, although the magnitude of this benefit has varied across treatment lines, endocrine partners, and patient subgroups [8,9,10,11,12,13,14].

As a result, CDK4/6 inhibitors are now widely used as part of standard treatment for most patients with HR-positive, HER2-negative metastatic breast cancer. Current guidelines generally favor their use early in the metastatic setting, particularly as first-line therapy in patients without visceral crisis [15,16]. However, recent European Society for Medical Oncology (ESMO) guidance and contemporary American Society of Clinical Oncology (ASCO) discussions on treatment sequencing have also acknowledged the implications of the SONIA trial, which challenged the necessity of universal upfront CDK4/6i use. In this trial, an upfront strategy of an aromatase inhibitor plus a CDK4/6 inhibitor followed by fulvestrant was compared with a deferred strategy of first-line aromatase inhibitor monotherapy followed by fulvestrant plus a CDK4/6 inhibitor at progression. Although upfront use was associated with improved PFS after two lines of therapy (PFS2), the updated SONIA analysis showed no overall survival advantage with first-line compared with second-line CDK4/6i use, while earlier use was associated with greater treatment exposure and toxicity, supporting a more individualized approach to treatment sequencing in selected patients [17,18,19,20,21]. These issues are especially relevant in real-world practice, where patients often differ from clinical trial populations in terms of age, comorbidity, disease tempo, treatment access, and treatment preference.

Despite the strong evidence supporting CDK4/6i, their optimal timing remains a clinically relevant question. In daily practice, some patients with endocrine sensitive, low volume, or indolent disease may achieve prolonged disease control with endocrine therapy alone before requiring combination treatment. For such patients, deferring CDK4/6i to the second-line setting may reduce early treatment exposure, toxicity, monitoring requirements, and cost, while preserving long-term disease control.

A key concern with deferred treatment is whether patients will remain fit enough to receive second-line therapy. Real-world attrition data suggest that this may be feasible for most patients with HR-positive, HER2-negative metastatic breast cancer. In a large real-world cohort, first-to-second-line attrition was 8.5% in this subgroup. Older age, shorter disease-free interval, and visceral involvement were associated with a higher risk of attrition [22]. These findings support the need for risk-adapted treatment sequencing rather than a uniform approach for all patients.

Real-world data remain limited on the clinical consequences of upfront versus deferred CDK4/6 inhibitor use, particularly with respect to overall survival. Although OS remains a key endpoint for evaluating treatment sequencing, real-world treatment decisions also depend on treatment burden, toxicity, monitoring requirements, cost, and access to subsequent therapies.

In this retrospective real-world study, we evaluated patients with HR-positive, HER2-negative metastatic breast cancer who received CDK4/6i as upfront or deferred treatment. We aimed to compare overall survival between first-line and second-line CDK4/6i use and to provide a clinically relevant perspective on treatment sequencing in daily practice.

## 2. Materials and Methods

### 2.1. Study Design and Patient Population

This retrospective real-world cohort study was conducted at two oncology centers in Türkiye: Ankara University Faculty of Medicine, Department of Medical Oncology, and Dr. Abdurrahman Yurtaslan Ankara Oncology Training and Research Hospital, Department of Medical Oncology.

The study included adult patients with HR-positive, HER2-negative metastatic breast cancer who received a CDK4/6 inhibitor between 1 January 2019 and 1 December 2024. Eligible patients had histologically confirmed breast cancer, documented metastatic disease, and received a CDK4/6 inhibitor either in the first-line or second-line metastatic setting.

Patients were grouped according to the timing of CDK4/6i initiation. Patients who received a CDK4/6i as part of first-line treatment for metastatic disease were defined as the upfront-use group. Patients who received systemic therapy without a CDK4/6i in the first-line setting and subsequently received a CDK4/6i in the second-line setting were defined as the deferred-use group. Patients who received CDK4/6i in the third or later treatment lines were excluded.

Clinicopathological and treatment data were obtained from electronic medical records. Collected variables included age at CDK4/6i initiation, menopausal status, Eastern Cooperative Oncology Group (ECOG) performance status, Charlson Comorbidity Index (CCI), de novo metastatic disease, visceral metastasis, endocrine sensitivity, year of metastatic diagnosis, CDK4/6i treatment line, CDK4/6i type, dose reduction, treatment-related adverse events, objective response, disease control, and vital status.

### 2.2. Ethics Approval

The study was approved by the Ankara University Human Research Ethics Committee on 19 December 2024, with the approval number İ11-856-24. The study was conducted in accordance with the principles of the Declaration of Helsinki. Given the retrospective design of the study, the requirement for informed consent was waived by the ethics committee.

### 2.3. Definitions

Hormone receptor positivity was defined as estrogen receptor and/or progesterone receptor positivity according to institutional pathology reports. HER2-negative disease was defined according to contemporary guideline-based pathological assessment.

Endocrine-sensitive disease was defined as no prior systemic treatment for metastatic disease or relapse occurring ≥12 months after completion of adjuvant endocrine therapy. Endocrine-resistant disease was defined as relapse during adjuvant endocrine therapy, relapse within 12 months after completion of adjuvant endocrine therapy, or progression during endocrine-based treatment for metastatic disease.

Visceral metastasis was defined as the presence of metastatic involvement in visceral organs, including liver, lung, brain, or other non-bone/non-lymph-node visceral sites. De novo metastatic disease was defined as metastatic disease present at initial breast cancer diagnosis.

Duration of CDK4/6 inhibitor treatment was assessed as a descriptive measure of treatment exposure. It was defined as the time from CDK4/6 inhibitor initiation to permanent discontinuation. For patients who were still receiving CDK4/6 inhibitor therapy at the last follow-up, treatment duration was calculated up to the date of last follow-up.

Objective response rate was defined as the proportion of patients who achieved complete or partial response as best response. Disease control rate was defined as the proportion of patients who achieved complete response, partial response, or stable disease. Response outcomes were assessed among patients with available response data.

### 2.4. Outcomes

The primary endpoint was OS, defined as the time from the date of metastatic disease diagnosis to death from any cause or last follow-up. An additional exploratory endpoint was PFS from CDK4/6 inhibitor initiation (CDK4/6i-PFS), defined as the time from CDK4/6 inhibitor initiation to disease progression or death, whichever occurred first. For patients in the upfront-use group, this corresponded to PFS during first-line CDK4/6 inhibitor therapy, whereas for patients in the deferred-use group, it represented PFS during second-line CDK4/6 inhibitor therapy.

Other secondary and descriptive outcomes included duration of CDK4/6 inhibitor treatment, dose reduction rate, treatment-related adverse events, objective response rate, and disease control rate.

### 2.5. Statistical Analysis

Descriptive statistics were used to summarize baseline characteristics, treatment exposure, toxicity, and response outcomes. Continuous variables were reported as median and interquartile range. Categorical variables were reported as frequencies and percentages. Between-group comparisons were performed using the Mann–Whitney U test for continuous variables and the chi-square test or Fisher’s exact test for categorical variables, as appropriate.

Overall survival and CDK4/6i-PFS were estimated using the Kaplan–Meier method, and survival curves were compared using the log-rank test. Median follow-up was estimated using the reverse Kaplan–Meier method.

Cox proportional hazards regression analysis was used to evaluate factors associated with overall survival. Univariable Cox regression analyses were performed for clinically relevant variables, including CDK4/6 inhibitor timing, ECOG performance status, visceral metastasis, Charlson Comorbidity Index, endocrine sensitivity, and year of metastatic diagnosis. A multivariable Cox regression model was then constructed to evaluate the independent association between CDK4/6 inhibitor timing and overall survival after adjustment for ECOG performance status, visceral metastasis, Charlson Comorbidity Index, endocrine sensitivity, and year of metastatic diagnosis. Year of metastatic diagnosis was included to account for changes in CDK4/6 inhibitor access, reimbursement, clinical practice, and follow-up maturity over time.

As a sensitivity analysis, inverse probability of treatment weighting (IPTW) was performed to reduce baseline differences between the upfront and deferred groups. Propensity scores (PSs) were estimated using logistic regression including year of metastatic diagnosis, endocrine sensitivity, visceral metastasis, ECOG performance status, and Charlson Comorbidity Index. Stabilized weights were calculated as P(deferred)/PS for patients in the deferred group and P(upfront)/(1−PS) for patients in the upfront group. Covariate balance before and after weighting was assessed using standardized mean differences. Effective sample size (ESS) was calculated after weighting. A weighted Cox proportional hazards model with robust standard errors was used to evaluate the association between CDK4/6 inhibitor timing and overall survival. Weight trimming at the 1st and 99th percentiles was also performed as an additional sensitivity analysis.

A 12-month landmark analysis was performed as a sensitivity analysis to address guarantee-time bias. This timepoint was selected as a pragmatic compromise between allowing sufficient time for patients to transition to second-line therapy and avoiding excessive loss of patients from the analysis, thereby preserving statistical power. Patients who died or were censored before 12 months after metastatic diagnosis were excluded. Overall survival was recalculated from the 12-month landmark time, and Cox regression analysis was repeated using the same covariates as in the primary multivariable model.

Hazard ratios (HRs) were reported with 95% confidence intervals (CIs). A two-sided *p* value of <0.05 was considered statistically significant. Analyses were performed using IBM SPSS Statistics for Windows, version 27.0 (IBM Corp., Armonk, NY, USA), and R version 4.4.2 (R Foundation for Statistical Computing, Vienna, Austria).

## 3. Results

### 3.1. Patient Characteristics

A total of 403 patients with HR-positive, HER2-negative metastatic breast cancer were included in the analysis. Of these, 311 patients received a CDK4/6 inhibitor in the first-line setting and were included in the upfront-use group, whereas 92 patients received a CDK4/6 inhibitor in the second-line setting and were included in the deferred-use group.

Baseline characteristics according to CDK4/6 inhibitor timing are summarized in Table 1. The median age at CDK4/6 inhibitor initiation was similar between the upfront-use and deferred-use groups (56.0 vs. 58.5 years; *p* = 0.171). Charlson Comorbidity Index, ECOG performance status, menopausal status, and de novo metastatic disease status were also comparable between groups. However, the deferred-use group had a less favorable disease profile, with higher frequencies of visceral metastasis (56.5% vs. 37.9%; *p* = 0.002) and endocrine-resistant disease (73.9% vs. 38.9%; *p* < 0.001).

### 3.2. Treatment Exposure, Toxicity, and Response

Treatment exposure, toxicity, and response outcomes are shown in Table 1. CDK4/6 inhibitor type differed significantly between groups. Ribociclib was more frequently used in the upfront-use group than in the deferred-use group (73.3% vs. 55.4%), whereas palbociclib use was more common in the deferred-use group (44.6% vs. 26.7%; *p* = 0.001).

Median duration of CDK4/6 inhibitor treatment was significantly longer in the upfront-use group than in the deferred-use group (21.0 vs. 13.5 months; *p* < 0.001). Dose reduction rates were similar between groups (36.0% vs. 41.3%; *p* = 0.356). Among patients with available toxicity data, treatment-related adverse event rates were also comparable (80.5% vs. 80.7%; *p* = 0.963).

Response data were available for 369 patients. Objective response rate was numerically higher in the upfront-use group, although the difference did not reach statistical significance (31.4% vs. 22.1%; *p* = 0.095). Disease control rate was significantly higher in the upfront-use group than in the deferred-use group (85.5% vs. 74.4%; *p* = 0.017).

### 3.3. Progression-Free Survival from CDK4/6 Inhibitor Initiation

During follow-up, 221 of 311 patients (71.1%) in the upfront-use group and 82 of 92 patients (89.1%) in the deferred-use group experienced a PFS event. Median CDK4/6i-PFS was 27.0 months (95% CI, 22.3–31.7) in the upfront-use group and 14.0 months (95% CI, 10.9–17.1) in the deferred-use group (log-rank *p* < 0.001).

### 3.4. Follow-Up and Overall Survival

At the time of analysis, death had occurred in 204 patients (50.6%) in the overall cohort. Death events were observed in 145 patients (46.6%) in the upfront-use group and in 59 patients (64.1%) in the deferred-use group.

**Figure 1:**
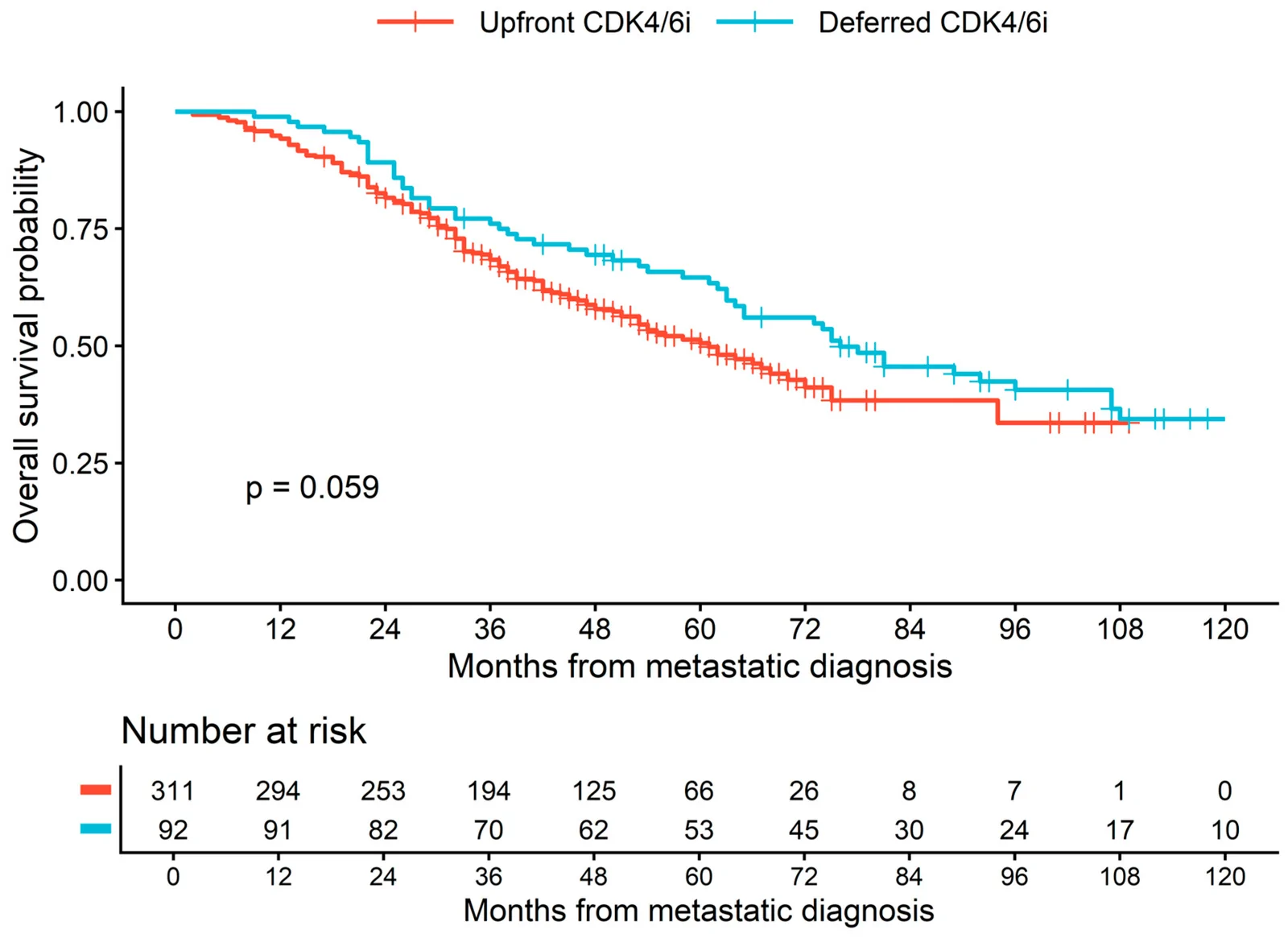
Kaplan–Meier curves for overall survival according to CDK4/6 inhibitor timing.

Median follow-up, estimated using the reverse Kaplan–Meier method, was 63.0 months in the overall cohort, 55.0 months in the upfront-use group, and 109.0 months in the deferred-use group. Median OS was 65.0 months in the overall cohort. Median OS was 61.0 months in the upfront-use group and 76.0 months in the deferred-use group. Although median OS was numerically longer in the deferred-use group, the difference did not reach statistical significance (log-rank *p* = 0.059; Figure 1). This finding should be interpreted in the context of the significantly longer follow-up duration in the deferred-use group.

### 3.5. Cox Regression Analysis for Overall Survival

Univariable and multivariable Cox regression analyses for OS are shown in Table 2. In univariable analysis, deferred CDK4/6 inhibitor use was not significantly associated with OS compared with upfront use (HR 0.725, 95% CI, 0.518–1.015; *p* = 0.061). ECOG ≥ 2, visceral metastasis, and year of metastatic diagnosis were associated with worse OS in univariable analysis.

In the multivariable Cox regression model adjusted for ECOG performance status, visceral metastasis, CCI, endocrine sensitivity, and year of metastatic diagnosis, deferred CDK4/6 inhibitor use was not independently associated with OS compared with upfront use (HR 1.167, 95% CI, 0.772–1.765; *p* = 0.463). ECOG ≥ 2 remained independently associated with worse OS (HR 2.765, 95% CI, 1.746–4.380; *p* < 0.001). Year of metastatic diagnosis also remained significantly associated with OS (HR 1.309, 95% CI, 1.193–1.436; *p* < 0.001). This variable was included to account for changes in CDK4/6 inhibitor access, reimbursement, clinical practice, and follow-up maturity over time, rather than as a biological prognostic factor.

In the IPTW analysis, baseline differences between the upfront and deferred groups were reduced, particularly for endocrine sensitivity and year of metastatic diagnosis. The effective sample sizes after weighting were 211.9 in the upfront group and 22.9 in the deferred group. In the weighted Cox regression model, deferred CDK4/6 inhibitor use was not significantly associated with OS compared with upfront use (HR 0.925, 95% CI, 0.552–1.549; *p* = 0.766). This finding was consistent in the weight-truncated sensitivity model (HR 0.973, 95% CI, 0.592–1.598; *p* = 0.913).

CDK4/6 inhibitor type differed between groups, with ribociclib being more frequently used in the upfront-use group and palbociclib being more common in the deferred-use group. However, CDK4/6 inhibitor type was not associated with OS in either univariable analysis (HR 1.014, 95% CI, 0.757–1.359; *p* = 0.926) or in a sensitivity multivariable model including CDK4/6 inhibitor type (HR 0.980, 95% CI, 0.715–1.343; *p* = 0.899).

In a 12-month landmark sensitivity analysis including 383 patients who were alive and under follow-up at 12 months after metastatic diagnosis, deferred CDK4/6 inhibitor use was not significantly associated with OS in either univariable analysis (HR 0.783, 95% CI, 0.554–1.105; *p* = 0.164) or multivariable analysis adjusted for the same covariates as the primary model (HR 1.347, 95% CI, 0.881–2.060; *p* = 0.168).

## 4. Discussion

In this two-center real-world cohort of patients with HR-positive, HER2-negative metastatic breast cancer, second-line deferred CDK4/6i use was not independently associated with inferior overall survival compared with upfront first-line use after adjustment for clinically relevant factors and calendar-period effects. Although median overall survival was numerically longer in the deferred-use group, this difference did not reach statistical significance in Kaplan–Meier analysis. These findings suggest that, in selected patients, deferring CDK4/6 inhibitor therapy to the second-line setting may not necessarily compromise overall survival. However, this result should be interpreted as hypothesis-generating and should not be considered evidence of equivalence or non-inferiority.

CDK4/6 inhibitors combined with endocrine therapy have become a standard treatment option in both first- and second-line settings, mainly because of the consistent progression-free survival benefit across pivotal randomized trials. However, most first-line registration trials did not include protocol-defined crossover to CDK4/6 inhibitors at progression. Therefore, these trials established the efficacy of CDK4/6 inhibition but did not directly answer whether upfront use improves long-term outcomes compared with planned deferred use [21,23]. This distinction is important in a disease course in which many patients receive multiple lines of therapy and where overall survival, treatment burden, toxicity, access, and patient preference all influence clinical decision-making.

The SONIA trial directly addressed this sequencing question in a randomized setting. Our study differs from SONIA in several important aspects. SONIA was conducted in a healthcare system with protocol-defined treatment sequencing and broad access to subsequent therapies. In contrast, our study reflects routine clinical practice in Türkiye, where CDK4/6 inhibitor timing was influenced by evolving drug availability, reimbursement status, and physician decision-making over time. In the earlier years of CDK4/6 inhibitor access, many patients had already received prior systemic therapy for metastatic disease before starting these agents, resulting in more frequent second-line use. As reimbursement became established and clinical experience increased, first-line CDK4/6 inhibitor use became more common. Therefore, treatment line in our cohort reflects not only disease biology and physician preference but also calendar period and treatment access. This temporal difference is also reflected in the substantially longer follow-up duration in the deferred-use group. Although year of metastatic diagnosis was included in the multivariable and propensity-weighted analyses, residual treatment-era confounding cannot be excluded. Similar calendar-period effects related to staggered drug approval and access were also reported in the POLiCDK study [24].

Furthermore, comparing upfront and deferred strategies in a retrospective setting may introduce guarantee-time bias, because patients in the deferred-use group must survive and remain clinically fit long enough to receive second-line CDK4/6 inhibitor therapy. To partially address this issue, we performed a 12-month landmark sensitivity analysis which showed no significant association between deferred CDK4/6 inhibitor use and overall survival. Nevertheless, residual guarantee-time bias cannot be fully excluded, and these findings should be interpreted as exploratory.

The deferred-use group had a less favorable disease profile, with higher rates of visceral metastasis and endocrine-resistant disease. These differences may have influenced prognosis independently of treatment sequencing. Although visceral metastasis and endocrine sensitivity were incorporated into the multivariable Cox and IPTW models, statistical adjustment can only account for measured covariates and cannot eliminate residual confounding from factors such as disease burden, disease tempo or physician treatment selection. Nevertheless, deferred CDK4/6 inhibitor use was not independently associated with worse OS after adjustment; ECOG performance status remained the strongest independent prognostic factor, whereas visceral metastasis was no longer statistically significant in the multivariable model.

Treatment exposure differed between groups, with a significantly longer median duration of CDK4/6 inhibitor therapy in the upfront-use group. This is expected, because first-line initiation exposes patients to CDK4/6 inhibition earlier and for a longer period. Similar observations were reported in SONIA and a recent multicenter retrospective study, although the latter included a smaller cohort and combined second- and later-line treatments [23,25]. In contrast, our analysis included a larger cohort and was restricted to first-line versus second-line CDK4/6 inhibitor use, thereby addressing a more specific and clinically relevant sequencing question.

PFS from CDK4/6 inhibitor initiation was longer in the upfront-use group, with a median of 27.0 months compared with 14.0 months in the deferred-use group. However, this finding should be interpreted in the context of treatment line, because CDK4/6i-PFS represents first-line PFS in the upfront group and second-line PFS in the deferred group. In addition, the deferred-use group had higher rates of endocrine-resistant disease and visceral metastasis. Therefore, this difference should not be interpreted as a direct comparison of sequencing strategies, although it is consistent with greater disease control during CDK4/6 inhibitor treatment when these agents are used earlier.

Response outcomes should also be interpreted carefully. In our cohort, disease control rate was higher in the upfront-use group, whereas objective response rate was numerically higher but did not reach statistical significance. This suggests that first-line CDK4/6 inhibitor use may provide better early disease control. In a broader context, a recent meta-analysis also suggested a potential advantage of first-line CDK4/6 inhibitor use in disease-control-related outcomes, reporting longer PFS2 in pooled analyses; however, this advantage was not maintained when analysis was restricted to randomized trials, and no clear overall survival difference was observed [26].

CDK4/6 inhibitor type differed between treatment groups in our cohort. Ribociclib was more frequently used in the upfront-use group, whereas palbociclib was more common in the deferred-use group. This imbalance likely reflects changes in clinical practice over time, including drug availability and the emergence of mature overall survival data from pivotal CDK4/6 inhibitor trials [9,11,14,27]. This point is relevant when interpreting our findings in relation to SONIA, in which most patients received palbociclib because ribociclib and abemaciclib became reimbursed later during the study period [23]. In contrast, our cohort included a higher proportion of patients treated with ribociclib, particularly in the upfront-use group. However, despite this imbalance in our cohort, CDK4/6 inhibitor type was not associated with overall survival in univariable analysis or in a sensitivity multivariable model.

The feasibility of deferred treatment depends on whether patients remain fit enough to receive second-line therapy. Although data are limited, real-world evidence suggests that first-to-second-line attrition is relatively low in hormone receptor-positive, HER2-negative metastatic breast cancer, with an attrition rate of 8.5% reported in the GIM14/BIO-META cohort [22]. However, because attrition risk may be higher in those with poor performance status, shorter disease-free interval, or visceral involvement, deferred use should not be applied indiscriminately.

The present study has several limitations. First, its retrospective design introduces the possibility of selection bias and residual confounding. Treatment allocation was not randomized and was influenced by evolving CDK4/6 inhibitor availability, reimbursement policies, and physician decision-making over time. Although year of metastatic diagnosis was included in the multivariable model, residual treatment-era confounding cannot be excluded.

Second, follow-up duration differed substantially between groups, and guarantee-time bias remains an important limitation. Although a 12-month landmark sensitivity analysis yielded results consistent with the primary analysis, residual bias cannot be fully excluded. Therefore, the survival findings should be interpreted cautiously and considered exploratory. First-line progression dates were not collected in the study dataset for patients in the deferred-use group, and therefore first-line PFS could not be assessed across the full cohort; the data required to calculate PFS2 were also not collected. Finally, molecular data, including PIK3CA, ESR1, and other resistance-related alterations, were not systematically available, and the two-center design may limit generalizability.

Despite these limitations, our study addresses a clinically relevant real-world question for which overall survival data remain limited. Unlike some previous retrospective cohorts that combined second- and later-line CDK4/6 inhibitor use or focused mainly on progression-based outcomes, our analysis was restricted to first-line versus second-line deferred use and evaluated overall survival in a relatively larger real-world cohort. The study also incorporated adjustment for calendar-period effects, which is particularly important in settings where CDK4/6 inhibitor access, reimbursement, and clinical adoption evolved over time.

These findings do not support routine deferral of CDK4/6 inhibitors for all patients, nor do they establish non-inferiority of deferred therapy. Rather, they support a risk-adapted and individualized sequencing approach. Upfront CDK4/6 inhibitor therapy remains appropriate for patients with high-risk features, symptomatic disease, rapid progression, high disease burden, visceral involvement, or concern for early attrition. In contrast, selected patients with endocrine-sensitive, indolent, low-volume disease and good performance status may be candidates for initial endocrine therapy alone with planned CDK4/6 inhibitor use at progression.

## 5. Conclusions

In conclusion, in this two-center real-world cohort of patients with hormone receptor-positive, HER2-negative metastatic breast cancer, second-line deferred CDK4/6 inhibitor use was not independently associated with inferior overall survival compared with upfront use after adjustment for year of metastatic diagnosis and clinically relevant prognostic factors. Our data support a risk-adapted and individualized approach and suggest that treatment sequencing may be reasonable in carefully selected patients.

## Figures and Tables

**Table 1:** Patient, treatment, toxicity, and response characteristics.

Characteristic	Upfront CDK4/6i n = 311	Deferred CDK4/6i n = 92	*p* Value
Age at CDK4/6i initiation, median (IQR), years	56.0 (48.0–65.0)	58.5 (48.8–67.8)	0.171
Charlson Comorbidity Index, median (IQR)	7.0 (6.0–8.0)	7.0 (6.0–8.0)	0.828
ECOG performance status, n (%)			0.274
ECOG 0–1	291 (93.6)	83 (90.2)	
ECOG ≥ 2	20 (6.4)	9 (9.8)	
Menopausal status, n/N (%)			0.462
Premenopausal	98 (31.5)	25 (27.5)	
Postmenopausal	213 (68.5)	66 (72.5)	
De novo metastatic disease, n (%)	146 (46.9)	37 (40.2)	0.255
Recurrent/metachronous metastatic disease, n (%)	165 (53.1)	55 (59.8)	
Visceral metastasis, n (%)	118 (37.9)	52 (56.5)	0.002
No visceral metastasis, n (%)	193 (62.1)	40 (43.5)	
Endocrine sensitivity, n (%)			<0.001
Endocrine-sensitive disease	190 (61.1)	24 (26.1)	
Endocrine-resistant disease	121 (38.9)	68 (73.9)	
CDK4/6 inhibitor type, n (%)			0.001
Ribociclib	228 (73.3)	51 (55.4)	
Palbociclib	83 (26.7)	41 (44.6)	
Endocrine partner, n (%)			<0.001
Letrozole	244 (78.5)	29 (31.5)	
Fulvestrant	67 (21.5)	63 (68.5)	
CDK4/6i–endocrine therapy combination, n (%)			<0.001
Ribociclib + letrozole	181 (58.2)	16 (17.4)	
Ribociclib + fulvestrant	47 (15.1)	35 (38)	
Palbociclib + letrozole	63 (20.3)	13 (14.1)	
Palbociclib + fulvestrant	20 (6.4)	28 (30.4)	
CDK4/6i duration of treatment, median (IQR), months	21.0 (11.0–33.0)	13.5 (6.0–25.3)	<0.001
Dose reduction, n/N (%)	112/311 (36.0)	38/92 (41.3)	0.356
Treatment-related adverse event, n/N (%)	227/282 (80.5)	67/83 (80.7)	0.963
Objective response rate, n/N (%)	89/283 (31.4)	19/86 (22.1)	0.095
Disease control rate, n/N (%)	242/283 (85.5)	64/86 (74.4)	0.017

Abbreviations: CDK4/6i, cyclin-dependent kinase 4/6 inhibitor; ECOG, Eastern Cooperative Oncology Group; IQR, interquartile range.

**Table 2:** Univariable and multivariable Cox regression analyses for overall survival.

Variable	Univariable HR (95% CI)	*p* Value	Multivariable HR (95% CI)	*p* Value
Deferred vs. Upfront CDK4/6i	0.725 (0.518–1.015)	0.061	1.167 (0.772–1.765)	0.463
ECOG ≥ 2 vs. 0–1	3.188 (2.058–4.937)	<0.001	2.765 (1.746–4.380)	<0.001
Visceral Metastasis (Present vs. Absent)	1.390 (1.055–1.832)	0.019	1.267 (0.950–1.690)	0.108
Charlson Comorbidity Index	1.079 (0.974–1.196)	0.146	1.070 (0.963–1.187)	0.208
Endocrine Resistance (vs. Sensitivity)	1.083 (0.819–1.431)	0.576	1.256 (0.933–1.691)	0.133
Year of Metastatic Diagnosis	1.261 (1.163–1.369)	<0.001	1.309 (1.193–1.436)	<0.001

Abbreviations: CDK4/6i, cyclin-dependent kinase 4/6 inhibitor; CI, confidence interval; ECOG, Eastern Cooperative Oncology Group; HR, hazard ratio.

## Data Availability

The data analyzed in this study are not publicly available because of patient confidentiality and national data-protection regulations. De-identified data may be made available from the corresponding author upon request, subject to institutional and ethics committee approval.

## Abbreviations

The following abbreviations are used in this manuscript:

ASCO	American Society of Clinical Oncology
CCI	Charlson Comorbidity Index
CDK4/6	Cyclin-dependent kinase 4 and 6
CDK4/6i	Cyclin-dependent kinase 4/6 inhibitor
CDK4/6i-PFS	Progression-free survival from CDK4/6 inhibitor initiation
CI	Confidence interval
ECOG	Eastern Cooperative Oncology Group
ESMO	European Society for Medical Oncology
ESS	Effective sample size
HER2	Human epidermal growth factor receptor 2
HR	Hazard ratio
HR-positive	Hormone receptor-positive
IPTW	Inverse probability of treatment weighting
IQR	Interquartile range
OS	Overall survival
PFS	Progression-free survival
PFS2	Progression-free survival after two lines of therapy
PS	Propensity score
